# A Real-Time Cascade Framework for UAV-Based Insulator Defect Detection with Attention-Guided Lightweight CNN

**DOI:** 10.3390/s26165126

**Published:** 2026-08-13

**Authors:** Zeliha Doğan Ersoy, Mustafa Gelmez, Durmuş Ersoy, M. Erdem Isenkul, Fırat Kaçar, Ali Ataş

**Affiliations:** 1 Department of Computer Engineering, Istanbul Esenyurt University, Istanbul 34510, Turkey; zelihadogan@esenyurt.edu.tr (Z.D.E.); durmusersoy@esenyurt.edu.tr (D.E.); 2 Department of Electric and Electronics Engineering, Istanbul University-Cerrahpasa, Istanbul 34310, Turkeyfkacar@iuc.edu.tr (F.K.); 3 Department of Research and Development, Mamur Technology Systems Inc., Istanbul 34590, Turkey; 4 Department of Computer Engineering, Istanbul University-Cerrahpasa, Istanbul 34310, Turkey; eisenkul@iuc.edu.tr; 5 Department of Motor Vehicles and Transportation Technologies, Istanbul Topkapi University, Istanbul 34020, Turkey

**Keywords:** deep learning, insulator fault detection, YOLO, MobileNetV2, CBAM, focal loss, dataset enhancement

## Abstract

**Highlights:**

**What are the main findings?**

- The proposed YOLO + MobileNetV2 cascade framework achieved 99.76% overall classification accuracy for insulator condition assessment.
- Integration of CBAM attention and Focal Loss improved classification performance, while the enriched dataset increased model generalization under real-field conditions.

**What are the implications of the main findings?**

- The proposed framework demonstrates that lightweight deep learning architectures can provide accurate and near real-time UAV-based transmission line inspection.
- Improved fault detection capability for broken and flashover insulators can support early maintenance decisions, reduce outage risks and improve power system reliability.

**Abstract:**

The physical condition of insulators on electrical transmission lines is critical for system reliability. Early fault detection such as broken discs or flashovers prevents outages and accidents. Traditional inspection methods are costly and difficult, driving demand for UAV-based autonomous systems. This article proposes a novel dataset enhancement method and a cascade deep learning model for detecting and classifying insulator disc conditions. Existing datasets were enriched by approximately 30% with wide-background, real-field images to improve model generalization. After comparative analyses, YOLO was selected for detection due to superior performance, while MobileNetV2 was chosen as the classifier for its processing speed advantage. CBAM attention module and Focal Loss function were integrated to boost classification performance and handle class imbalance. The resulting YOLO + MobileNetV2 model achieved 99.76% overall accuracy across “Normal”, “Broken”, and “Flashover” classes, with an F1-score of 0.99 for broken discs, operating with 185.60 ms latency, confirming its viability for real-time inspection.

## 1. Introduction

Monitoring transmission lines is critical for the stable operation of the power grid [1]. As fundamental components of these lines, insulators are consistently exposed to harsh environmental conditions, increasing their susceptibility to failures that can severely compromise grid stability. Because conductors in power systems maintain high potential differences relative to one another and their supporting structures, high-voltage insulators are essential. Mechanically, they must exhibit robust strength and weather resistance. Electrically, they must provide superior insulation to safely separate conductors, withstanding not only standard operating voltages but also extreme electrical stresses caused by lightning strikes or switching transients [2].

Traditional inspection methods are heavily reliant on manual labor, often requiring personnel to navigate treacherous mountainous terrains under extreme weather conditions. Consequently, these approaches are not only hazardous and time-consuming but also highly prone to human error and incomplete assessments. To address these limitations, Unmanned Aerial Vehicles (UAVs) have recently emerged as a highly flexible and efficient alternative for inspecting electrical infrastructure [1]. Equipped with high-resolution cameras, UAVs can easily traverse complex topographies to capture detailed images of insulators [2,3].

Furthermore, the rapid advancement of deep learning has revolutionized the automated analysis of these UAV-captured images [4,5]. By leveraging models such as Convolutional Neural Networks (CNNs), specific insulator defects including cracks and flashover marks can be automatically detected and classified. The integration of deep learning with UAV technology not only enhances diagnostic accuracy but also significantly accelerates processing speeds, enabling the real-time analysis of massive datasets during field operations.

## 2. Literature Review

Insulators in overhead power lines are highly vulnerable to environmental and mechanical degradation, necessitating rapid and accurate diagnostic methods to prevent severe power disruptions and economic losses. Porcelain, glass, and polymer insulators—especially modular cap-and-pin strings—frequently suffer from manufacturing defects, contamination, corrosion, and physical damage such as disc cracking, core fragmentation, or pollution-induced flashovers.

To assess the condition of transmission lines, operators traditionally rely on visual inspections conducted using binoculars, helicopters, or UAVs [6]. While visual checks, alongside infrared thermography [7], ultraviolet (UV) imaging [8], and electric field testing [9], provide valuable diagnostic data, their success heavily depends on the observation angle, equipment constraints, and manual interpretation. To automate this process, early studies utilized feature-based machine learning, combining color segmentation, edge detection, or wavelet transforms with Support Vector Machines (SVMs) [10]. However, these conventional approaches require extensive processing times due to the use of sliding-window strategies and rely heavily on manual feature extraction, limiting their scalability [11].

Deep learning has largely superseded these traditional methods by autonomously extracting complex features from UAV imagery [12]. Architectures like Faster R-CNN have demonstrated robust performance, achieving up to 97.6% accuracy in broken insulator detection [13]. Nevertheless, R-CNN-based frameworks are inherently hindered by slower inference speeds, making real-time UAV deployment challenging [14].

To address the complexities of cluttered aerial backgrounds, researchers have increasingly explored two-stage architectures. These frameworks decouple the detection process by first extracting insulator-specific regions of interest (ROIs) and subsequently applying localized anomaly detection or patch-segmentation algorithms. While two-stage models effectively suppress background noise and enhance the visibility of subtle defects, they typically introduce high computational overhead, massive parameter counts, and cascaded error propagation. These drawbacks significantly restrict their viability for real-time deployment on resource-constrained edge devices.

Two-stage detection and classification frameworks have been extensively investigated for insulator fault diagnosis due to their ability to exploit fine-grained feature representations and contextual information. Zhang et al. [15] proposed a framework that first localizes insulators using Oriented R-CNN and subsequently performs defect recognition through a Transformer-based Sequence Transduction module. Although the proposed architecture effectively captures long-range contextual dependencies, the combination of a ResNet-50 backbone and Multi-Head Self-Attention (MHSA) modules substantially increases computational complexity, limiting its applicability to real-time edge deployment. Similarly, Han et al. [16] introduced the STID framework, a two-stage cascaded architecture that combines foreground masking with reverse distillation for anomaly detection. While the framework effectively suppresses background interference, its cascaded design propagates segmentation errors from the first stage to the subsequent anomaly detection stage, thereby reducing overall robustness. Sun et al. [17] proposed a hybrid framework that integrates YOLOv5 with conventional image enhancement techniques, including gamma correction and image sharpening filters, to improve defect visibility under low-light conditions. Although these preprocessing operations enhance feature contrast, they introduce additional computational overhead and may reduce generalization under varying environmental conditions. Overall, despite their high detection accuracy, two-stage and hybrid detection–classification frameworks generally suffer from increased computational complexity, sequential error propagation, and limited suitability for resource-constrained embedded platforms.

Despite these algorithmic advancements, the datasets utilized in the literature present significant variability and limitations. Existing datasets [18,19] often suffer from data redundancy and constrained background diversity, focusing primarily on specific insulator types or relying on computer-generated synthetic defects. Other works [20,21] provide robust physical categorizations (e.g., broken, flashover, or specific contamination types) but are either not fully public or heavily dependent on controlled laboratory environments. This highlights a critical ongoing need for diverse, real-world benchmarks to accurately validate modern diagnostic frameworks.

## 3. Materials and Method

This section details the proposed methodology and materials for the autonomous inspection of power transmission line insulators using aerial imagery. A deep learning-based, two-stage architecture is introduced to detect insulator strings and assess the health status of individual discs. This system is specifically designed to preserve fine details in wide-angle, high-resolution images, thereby enhancing overall classification performance.

**Figure 1:**
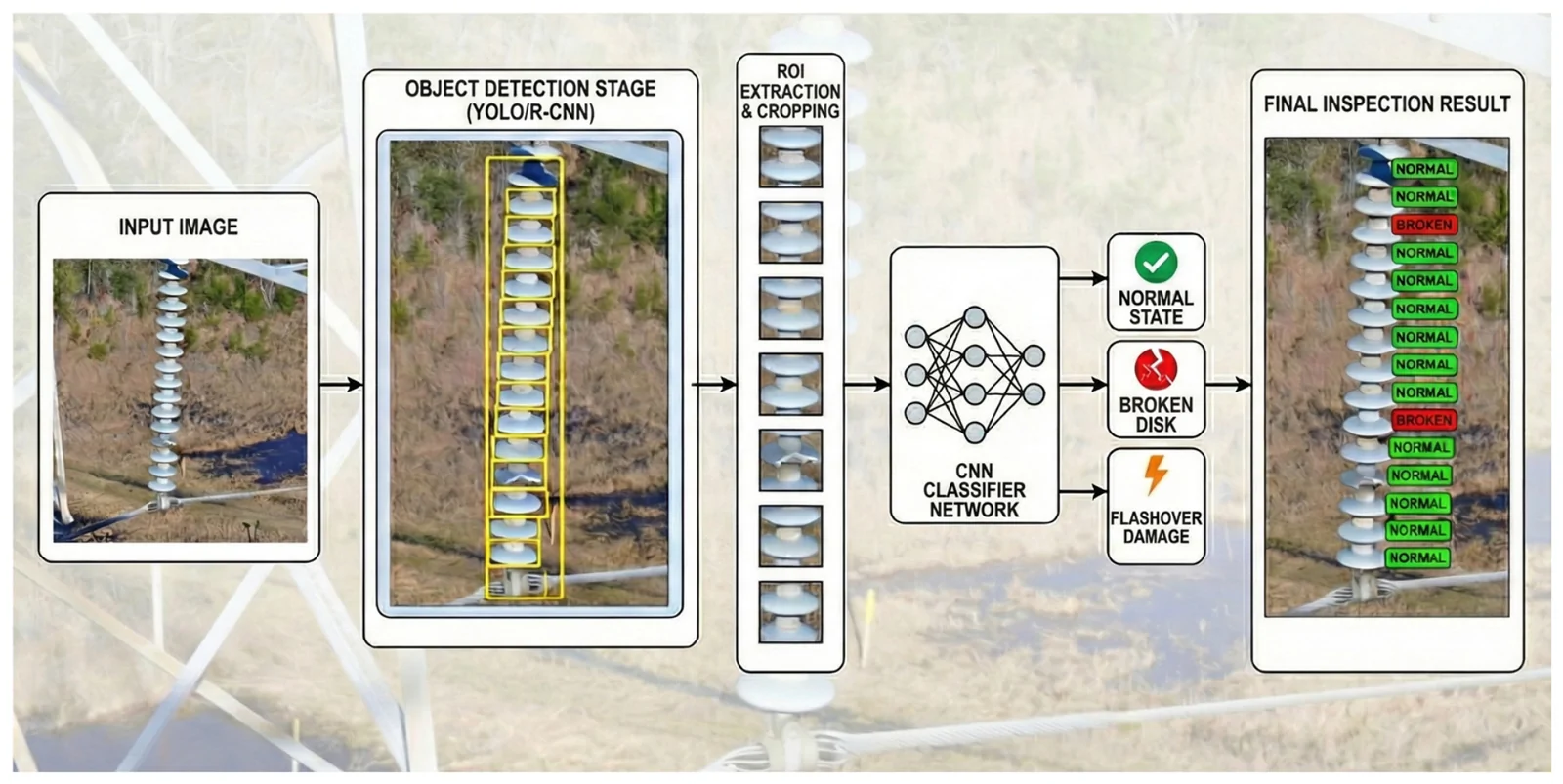
Block diagram detection and classification model.

In aerial images captured by UAVs or helicopters, insulator discs typically occupy a minimal portion of the entire field of view. Consequently, single-stage detection algorithms often struggle to identify subtle defects, such as capillary cracks or minor flashovers. To address this limitation, this study adopts a hierarchical approach that decouples the detection and classification processes. The proposed model, illustrated in Figure 1, comprises three sequential modules: object detection, region of interest (ROI) extraction, and deep classification. The system processes raw images by first localizing the target objects, isolating them, and subsequently performing a detailed condition analysis.

The system’s operational pipeline consists of sequential processes that transform raw data into final diagnostic decisions. Initially, high-resolution aerial photographs obtained during field inspections are fed into the system. These input images are then pre-processed to align with the dimensional requirements of the model’s training parameters.

The object detection module, serving as the primary stage of the workflow, is responsible for identifying the insulator strings and their constituent discs. Leveraging state-of-the-art object detection algorithms, this module determines the precise spatial coordinates of all discs within the image. Notably, the objective of this initial network is strictly localization, detecting the presence and position of the discs rather than evaluating whether they are normal or damaged. For each detected disc, the algorithm generates a bounding box and forwards these spatial coordinates to the subsequent extraction and classification stages.

Following the detection phase, ROI extraction is executed using the acquired spatial coordinates. During this process, each detected insulator disc is extracted from the primary image, effectively isolating it from background clutter and environmental noise. Consequently, rather than processing the entire large-scale image, the system generates a series of localized image patches focused entirely on the surface details of individual discs. This targeted approach mitigates the processing of extraneous data, enabling the classification network to concentrate exclusively on assessing the physical condition of the insulators.

These cropped ROI images are subsequently fed into the final stage: a deep learning-based classifier. Utilizing CNN architectures, this module extracts complex features from each patch to evaluate the disc’s current state. The network categorizes each analyzed image into one of three predefined classes: normal, broken, or flashover damage. The final output of the proposed model is presented by overlaying these diagnostic classifications as visual results directly onto the original raw image.

### 3.1. Dataset Creation and Properties

**Figure 2:**
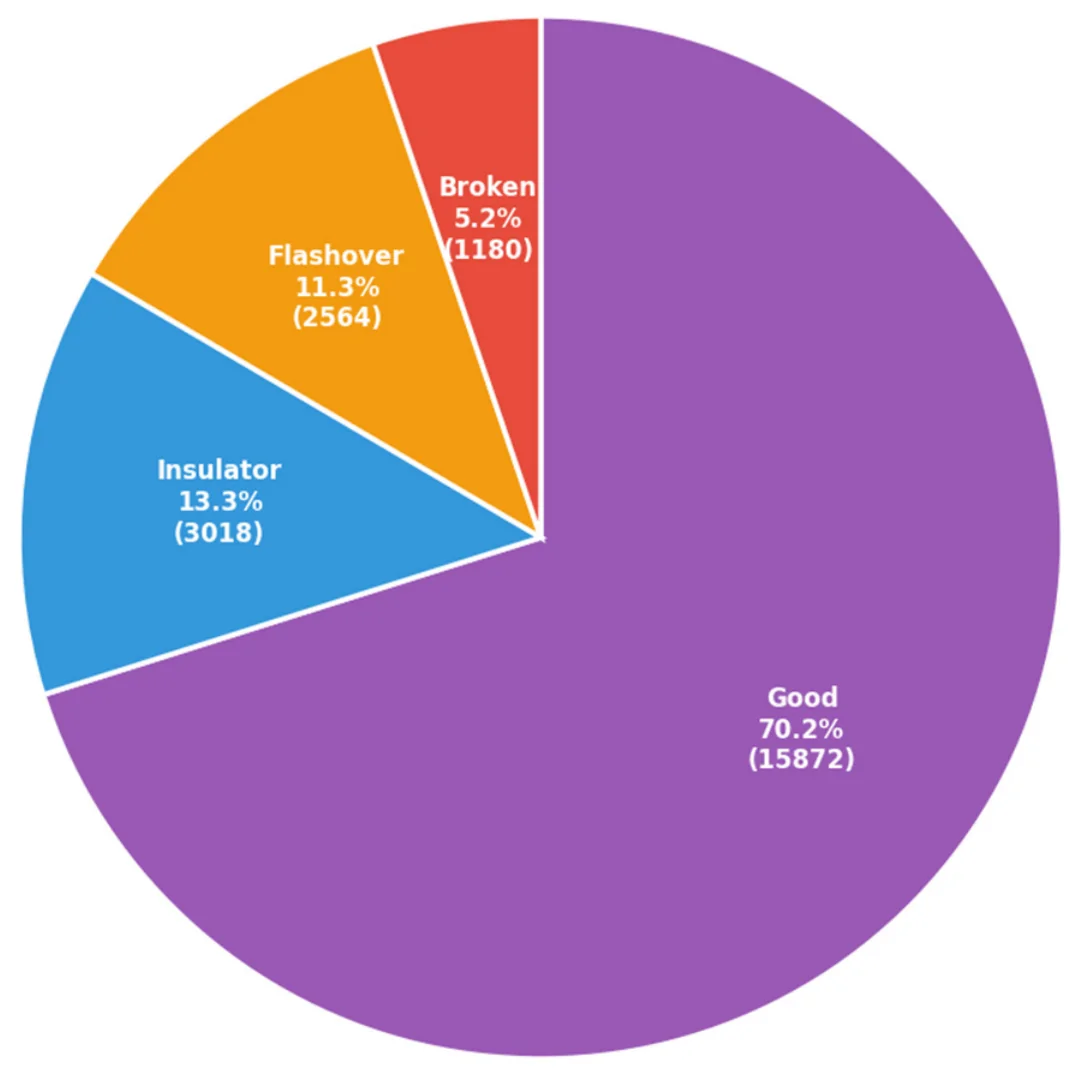
Class Distribution of the Proposed Dataset.

**Figure 3:**
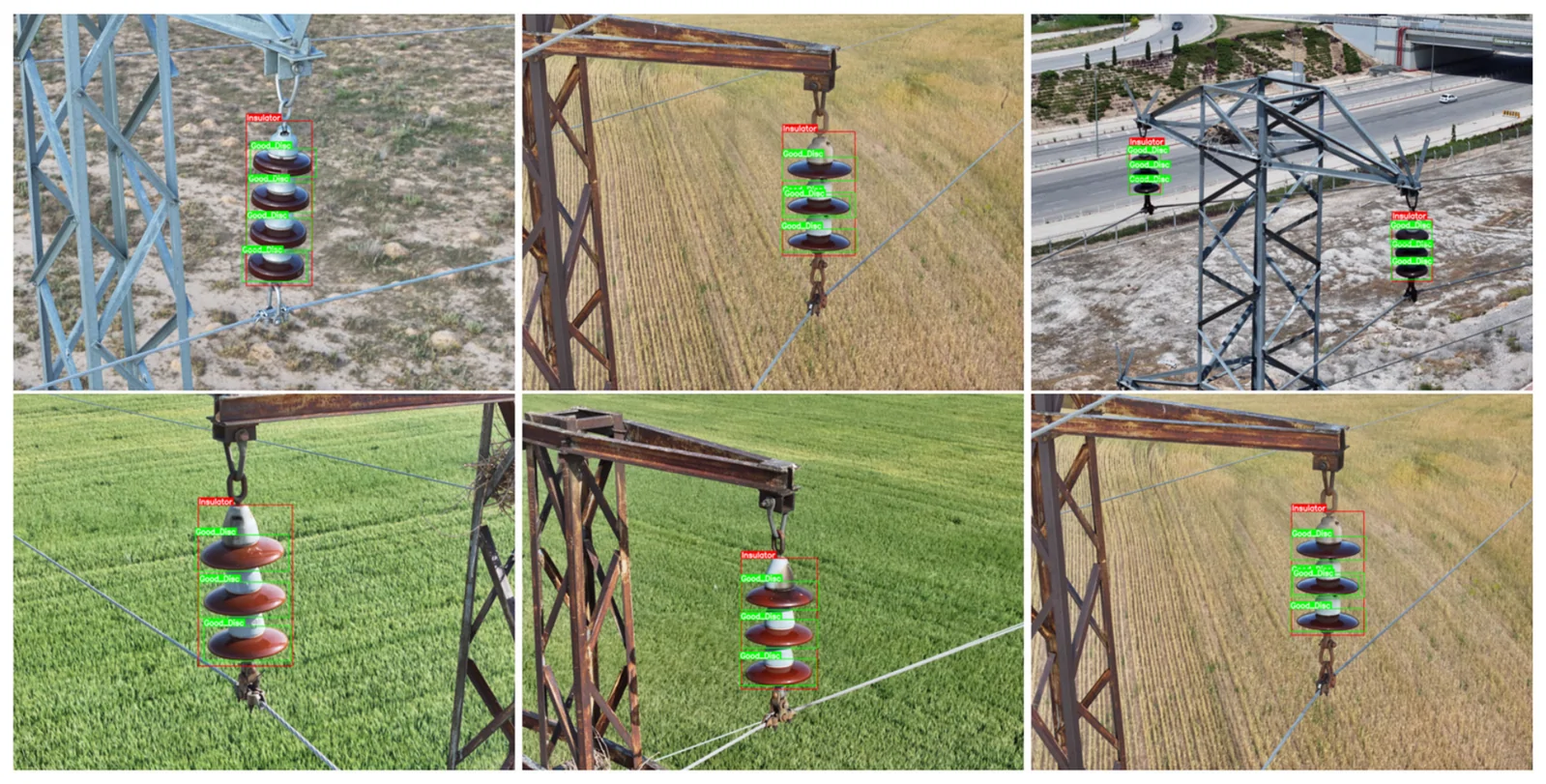
Labelled hybrid dataset examples with bounding boxes.

The quality and diversity of the dataset are critical factors influencing the performance and generalization capability of deep learning models. Because commonly used open-source datasets often fail to adequately capture the complexity of real-world conditions, this study introduces a hybrid dataset combining existing literature data with original field imagery. This approach is designed to enhance the model’s robustness in practical scenarios. The overall composition and distribution of the proposed hybrid dataset are illustrated in Figure 2. Representative examples of the labelled hybrid dataset with bounding boxes are presented in Figure 3. Field images were acquired using an UAV equipped with a DJI FC4382 camera sensor. To achieve a high-resolution output of 8064 × 6048 pixels (96 dpi), the camera was configured with a 19.35 mm focal length, an f/2.8 aperture, and a 1/250 s shutter speed. By combining the rare fault cases from the literature with environmentally diverse field images containing complex backgrounds, the final hybrid dataset closely mirrors the highly variable conditions of real-world UAV-based power line inspections. This comprehensive representation enhances the robustness of the trained model and its applicability to practical field deployments. The foundational dataset, presented by Lewis et al. [20], comprises 1600 images sourced from the literature. This reference dataset is crucial for providing the damaged examples necessary for the network to learn anomaly features. Annotation of this dataset yielded a total of 18,912 labeled objects. The class distribution consists of 13,364 normal discs, 1180 broken discs, 2564 discs with flashover damage, and 1804 insulator strings. Consequently, this dataset serves as the primary source for introducing rare fault classes (broken and flashover) into the training process. To improve the model’s adaptability to real-world environments and increase the variance within the normal class, a dedicated field data collection process was conducted. This effort yielded 359 high-resolution images from medium-voltage transmission poles composed of diverse materials, specifically 67 iron and 37 concrete poles. Within these images, 3722 objects were annotated. To minimize false-positive rates and ensure the network could reliably identify ideal insulator structures against complex backgrounds, all field data consisted strictly of healthy, undamaged samples. Thus, the field-collected dataset contributes 2508 normal disc labels and 1214 insulator string labels.

### 3.2. Comparison Object Detection Models

This study comparatively evaluates two fundamental deep learning paradigms prevalent in the literature for insulator detection and damage classification from aerial imagery: the single-stage YOLO architecture and the two-stage Faster R-CNN framework. The primary objective is to empirically determine which architecture optimally balances the dual requirements of high-precision detection and real-time operational capability.

Single-stage models like YOLO are renowned for their high-speed, single-pass object detection capabilities. However, the literature indicates that this speed-centric design traditionally entails specific trade-offs. Earlier YOLO iterations often exhibited limitations in detecting densely clustered or extremely small objects [22,23]. Furthermore, in scenarios involving complex backgrounds and low contrast, the localization accuracy of single-stage models can underperform relative to that of their two-stage counterparts [24]. Additionally, challenging lighting conditions such as reflective surfaces and glare can induce deviations in YOLO’s bounding box predictions [25].

Conversely, the two-stage Faster R-CNN architecture historically yields higher detection accuracy by decoupling region proposal from classification. Yet, this structural complexity significantly increases inference time, rendering the system suboptimal for real-time applications [25]. The candidate region proposals are generated by the Region Proposal Network (RPN). The first stage of the framework is susceptible to errors caused by complex backgrounds, occlusion, or inadequate lighting. These preliminary errors inevitably propagate to the subsequent classification stage [24]. The distribution of the hybrid dataset, including the literature-based and field-collected data, is summarized in Table 1.

Moreover, the substantial computational and memory demands of Faster R-CNN inherently restrict its efficient deployment on UAV-based embedded systems [25].

While object detection architectures are frequently evaluated along a trade-off spectrum between speed and accuracy, critical applications like autonomous insulator inspection demand both. Empirical tests conducted in this study revealed that Faster R-CNN-based models failed to meet real-time operational thresholds achieving less than 10 FPS and ultimately lagged behind the YOLO series in detection accuracy. In contrast, all evaluated YOLO variants (v11 and v12) achieved an AP50 score of 0.98, indicating a performance saturation point regarding accuracy. Consequently, inference speed and computational efficiency emerged as the decisive selection criteria. YOLOv12n was selected as the optimal architecture for rapid, precise field detection; despite achieving the same baseline accuracy as heavier models, it operates approximately 2.5 times faster while maintaining a low computational load and utilizing a state-of-the-art topological structure.

### 3.3. Object Detection YOLOv12 Architecture

The first step in the proposed hierarchical system is the precise determination of the positions of the insulator arrays and insulator discs within the image. At this stage, the YOLOv12n variation, which is the lightest version of the YOLOv12 architecture optimized for mobile devices, was used due to its real-time detection performance and low hardware resource consumption.

YOLOv12 builds upon the success of previous versions (v8/v11) but incorporates a ‘Cross-Stage Partial’ (CSP) backbone architecture that improves feature extraction and gradient flow, along with an advanced ‘Path Aggregation Network’ (PANet) neck design that better captures multi-scale objects. The model processes the image in a single forward pass, directly predicting object candidates and class probabilities using an anchor-free approach. This structure accelerates overlapping operations, particularly in detecting objects that are dense and congested in the image, such as insulator discs.

One of the most critical methods adopted in the detection phase of this study is the reduction of class complexity. In the original dataset, the discs are divided into three different subclasses: “Normal”, “Broken” and “Flashover”. However, forcing the detection model to find very small objects and classify them according to fine details at the same time can reduce detection performance. To overcome this problem, a class reduction process was applied prior to training the detection network. The Normal, Broken, and Flashover classes were merged under a single ‘Disc’ class (Class 0); “Insulator” was retained as a separate class (Class 1). Thus, the YOLOv12 network was not tasked with determining whether a disc was damaged; it focused solely on locating the disc. This strategy increased the stability of the detection model and ensured that more accurate coordinates were passed to the classifier in the second stage.

### 3.4. Comparison of Classification Models

In the second phase of the proposed hybrid architecture, the aim is to determine the health status of the detected insulator discs. In this phase, regions of interest extracted from the image are classified. To determine the most efficient classification backbone, three state-of-the-art deep CNN architectures with varying architectural depths, parameter counts, and memory requirements were comparatively analyzed.

ResNet-18 is a relatively lightweight variant of the ResNet architecture, which is widely used in industrial applications. It reduces the gradient vanishing problem and enables deeper networks to be trained more stably by adding residual connections to sequential convolutional layers. The architecture consists of residual blocks, each containing two convolutional layers and an identity mapping [24].

EfficientNet-B0 is a convolutional neural network architecture that utilises mobile back-convolutional residual blocks (MBConv) as its fundamental building blocks and jointly optimizes the network’s depth, width, and input resolution under a single composite scaling strategy. This approach enables high representational power with a limited number of parameters [24].

MobileNetV2 is a lightweight network architecture designed specifically for mobile devices and embedded systems. It reduces computational costs through deeply separable convolutions and combines inverted residuals with linear bottleneck blocks. This block structure offers significant advantages for applications requiring low latency and energy efficiency. These models were initialized with pre-trained weights on ImageNet and trained on the hybrid dataset. The results of the performance tests conducted on Tesla T4 GPU hardware are presented in Table 2.

When examining the classification performance of the models, EfficientNet-B0 achieved the highest accuracy rate at 99.51%. However, speed performance shows a sharp distinction depending on the hardware used. In the Tesla T4 GPU environment with high processing power, the ResNet-18 architecture processed 252.49 frames per second (FPS), making it the fastest model; however, it exhibited the lowest accuracy at 98.38%.

However, the situation changes in tests on the Intel i7-8750H processor (CPU). Thanks to its lightweight architecture, MobileNetV2 processes 22.31 frames per second on the CPU, outperforming both EfficientNet-B0 and ResNet-18 to become the fastest model for processor-based inference. MobileNetV2 follows the accuracy leader EfficientNet-B0 by a negligible difference of only 0.35% (99.16%).

In UAVs and embedded systems, the most limited resources are memory and storage space. Examining the table, MobileNetV2 has only 2.23 million parameters and occupies 8.81 MB of disc space. These values represent approximately 2 times less resource consumption compared to its closest competitor, EfficientNet-B0, and approximately 5 times less compared to ResNet-18.

This work aims to develop an autonomous system that can operate on platforms with limited hardware resources, such as UAVs. Although EfficientNet-B0 provides a marginal accuracy advantage, MobileNetV2’s 44% storage savings and its approximately 37% higher processing speed compared to its competitors on the CPU make it the most suitable option for embedded system integration. Therefore, the MobileNetV2 architecture, which offers the best balance between accuracy, storage efficiency, and processing speed, has been selected as the classification backbone of the system. To close the small accuracy gap where the standard MobileNetV2 lags behind EfficientNet-B0 and to improve its performance on challenging examples, the model has been customized by integrating the Convolutional Block Attention Module (CBAM).

### 3.5. Classification & Feature Extraction

To perform a detailed health status analysis of the detected insulator discs, a customized deep classification network was designed in the second phase of the proposed system. The MobileNetV2 architecture, selected based on the comparative analyses detailed in Section 3.4, was used as the backbone of this phase. However, to enhance the performance of the standard MobileNetV2 architecture in complex field conditions and to manage class imbalance in the dataset, two significant original contributions were made to the system: the integration of the Convolutional Block Attention Module (CBAM) and the use of the Focal Loss function.

The proposed classification model is based on an attention mechanism placed between the last convolutional layer of MobileNetV2 and the classifier layer. This structure enables the network to focus not only on the general shape of the disc but also on the fine details that determine the damage (break line, burn mark, etc.). The CBAM refines the incoming feature map (*F*) by passing it through two sequential sub-modules: Channel Attention and Spatial Attention. The Channel Attention Module (*Mc*) seeks to answer the question, ‘Which feature channel is more important?’ In the architecture applied to this model, both Global Average Pooling (AvgPool) and Global MaxPool operations are applied in parallel on the input features. The resulting vectors are passed through a shared Multi Layer Perceptron (MLP) network, and the results are aggregated and fed into a Sigmoid activation function. This process is mathematically expressed as follows:

The channel weights obtained here are multiplied by the input features to obtain refined features on a channel basis.

Spatial Attention Module ($M_{S}$): This module focuses on spatial localization, fundamentally determining where the salient features or defects are situated within the image. Using the feature map previously refined by the channel attention process, two 2D spatial maps are generated by applying AvgPool and MaxPool operations across the channel axis. These resulting maps are subsequently concatenated and processed through a 7 × 7 convolutional layer:

(1) $$M_{s}\left(F\right)=\sigma \left(f^{7\times 7}\left(\left[\mathrm{AvgPool}\left(F\right);\mathrm{MaxPool}\left(F\right)\right]\right)\right)$$

When the dataset analysis in Section 3.1 is examined, it is seen that the number of examples in the “Normal” class is higher than in the “Broken” and “Flashover” classes. When using Standard Cross-Entropy loss, it is likely that the model will achieve high accuracy by focusing on the majority class (Normal) but may miss critical errors that occur infrequently (Low Recall).

To overcome this problem, the Focal Loss function has been integrated into the training process. Applied to the model with parameters γ=2 and α=1, this function reduces the loss value of examples that the model classifies easily while emphasizing the loss of misclassified or difficult examples. The mathematical form of the Focal Loss function is as follows:

(2) $$FL\left(p_{t}\right)=-\alpha {\left(1-p_{t}\right)}^{\gamma }\log\left(p_{t}\right)$$

Here, $p_{t}$ is the probability value predicted by the model for the correct class. The term ${\left(1-p_{t}\right)}^{\gamma }$ dampens the effect of easy examples, directing the network’s learning capacity towards difficult examples. During the training process, the Weighted Random Sampling method was also used to physically balance the imbalance in the dataset. By assigning weight inversely proportional to the number of examples to each class, the representation of minority classes in each training batch was guaranteed.

### 3.6. Proposed Hybrid Decision Algorithm and End-to-End Integration

**Figure 4:**
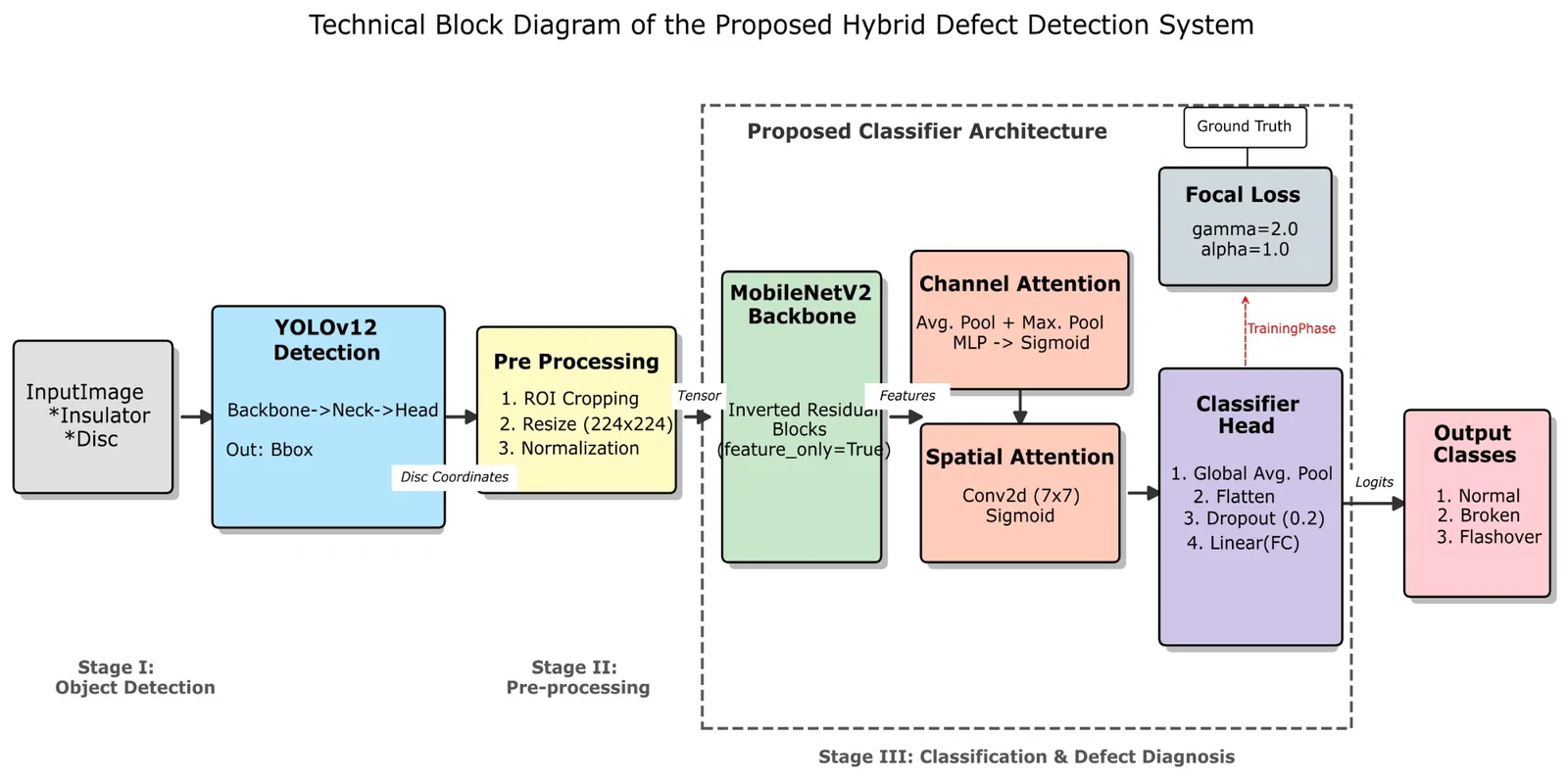
Block diagram of the proposed hybrid fault detection system. The asterisk (*) indicates the objects targeted for detection and classification by the proposed model.

One of the most original aspects of the system developed in this study is that the object detection and state classification processes are integrated within a complementary hierarchical system decision mechanism, rather than operating independently of each other. This approach aims to maximize error detection accuracy while reducing the system’s computational cost. The system operates through two fundamental sub-processes, detailed below, in accordance with the block diagram presented in Figure 4.

The bounding box coordinates $B=[x_{1},y_{1},x_{2},y_{2}]$ generated by YOLOv12n undergo the following preprocessing steps before being fed to the classifier in the second stage:

- Boundary Check: It is verified that the coordinates do not extend beyond the image boundaries ($x_{1}\geq 0,y_{1}\geq 0$).
- Cropping: Only the portion of the original image containing the relevant object is extracted.
- Scaling: Although the cropped images may have different dimensions depending on the distance from the camera, MobileNetV2 expects a fixed input. Therefore, the images are resized to 224 × 224 pixels using Bilinear Interpolation, preserving the details.
- Normalization: The color statistics of the ImageNet dataset are used to improve the model’s performance. For each RGB channel, the mean values [0.485, 0.456, 0.406] are subtracted and divided by the standard deviation values [0.229, 0.224, 0.225] to bring the data into the range expected by the model.

The system’s final output is governed by a conditional processing pipeline dependent on the detected object class. This hierarchical flow optimizes computational efficiency by preventing macroscopic structures, such as entire insulator strings, from being unnecessarily processed by the secondary classifier. Specifically, if the primary YOLO network identifies an object as an insulator string (Class ID: 1), the object bypasses the damage analysis phase entirely and is directly annotated with the “insulator” label. Conversely, if the detected object is an individual disc (Class ID: 0), the system triggers the secondary stage to evaluate its physical condition. The extracted disc image patch is forwarded to the trained Attention-MobileNetV2 network, which computes probability scores (*P*) across three predefined condition classes: normal, broken, and flashover damage. The final diagnostic state of the disc is determined by the class exhibiting the maximum probability (argmax(P)).

The localization efficacy of the YOLOv12n network, functioning as the primary detection stage, is quantified using the Intersection over Union (IoU) metric. The relationship between the predicted bounding box $\left(B_{pred}\right)$ and the ground truth bounding box ($B_{gt}$) is formulated as follows:

(3) $$IoU\left(B_{pred},B_{gt}\right)=Area\left(B_{pred}\cap B_{gt}\right)/Area\left(B_{pred}\cup B_{gt}\right)$$

Given the densely packed structure of the insulator strings, a specific spatial threshold, IoU≥0.5, was established for a detection to be considered valid. Consequently, predictions yielding an Intersection over Union (IoU) value below this threshold are categorically designated as false detections, regardless of the accuracy of the subsequent classification. Unlike standard classification paradigms, the components of the confusion matrix in this study are formulated in an integrated manner to encapsulate both localization and classification errors. Let $C_{gt}$ denote the ground truth class of the object and $C_{pred}$ denote the class predicted by the system. Within this integrated framework, a True Positive (TP) represents the optimal outcome where the system successfully localizes the object (IoU≥0.5) and accurately predicts its correct condition ($C_{pred}=C_{gt}$). Conversely, a False Positive (FP) represents a false alarm, which encompasses two distinct failure scenarios. The first scenario is a localization error, occurring either when background noise is erroneously identified as an object or when the spatial overlap fails to meet the threshold (IoU<0.5). The second scenario is a classification error, occurring when the object is adequately localized but assigned an incorrect label ($C_{pred}\neq C_{gt}$).

(4) $$FP=\{x|\left(IoU<0.5\right)\vee \left(\left(IoU≧0.5\right)\wedge \left(C_{pred}\neq C_{gt}\right)\right)\}$$

Finally, a False Negative (FN) occurs when an actual object present in the field of view remains entirely undetected by the system. From an operational and safety perspective, this constitutes the most critical error modality; overlooking a defective insulator disc could result in severe infrastructure failures.

Utilizing these integrated confusion matrix components, the overall diagnostic sensitivity and reliability of the proposed system are quantified through standard performance metrics. The primary metric evaluated is Precision, which assesses the reliability of the positive detections generated by the system. In practical field applications, maintaining high precision is paramount to ensure that “broken” or “flashover damage” alerts do not manifest as false alarms, thereby mitigating the financial and logistical costs associated with unnecessary maintenance operations. Precision is mathematically formulated as follows:

(5) $$Precision=\frac{TP}{TP+FP}$$

Recall: This metric indicates the proportion of actual field defects successfully identified by the system. From the perspective of energy transmission infrastructure security, maximizing this metric is critical to ensure that no damaged insulator discs are overlooked.

(6) $$Recall=\frac{TP}{TP+FN}$$

F1-Score: Given the inherent class imbalance within the dataset, relying solely on standard accuracy metrics can yield misleading interpretations. Therefore, the F1-Score, defined as the harmonic mean of Precision and Recall, is utilized to rigorously evaluate the model’s balanced performance.

(7) $$F1=2\times \frac{Precision\times Recall}{Precision+Recall}$$

Average Precision (mAP@50): To comprehensively assess the efficacy of the initial object detection phase, the arithmetic mean of the Average Precision (AP) values calculated across all classes is utilized. This metric indicates the model’s overall detection quality at an IoU threshold of 0.50. The following performance criteria were used to verify the applicability of the developed system in resource-constrained embedded platforms, such as UAVs:

Latency: This defines the total temporal delay from the input of a raw image to the generation of the final diagnostic decision. In a hybrid structure, this time is equal to the sum of the detection time ($t_{det}$), cropping time ($t_{crop}$), and classification time ($t_{cls}$):

(8) $$\mathrm{Latency}=t_{det}+t_{crop}+t_{cls}$$

Frames Per Second (FPS): This represents the processing throughput, indicating the number of images the system can process in a unit of time. It is mathematically expressed as the inverse of the total latency period.

## 4. Results

This section presents the experimental results of the proposed hybrid deep learning architecture, integrating YOLOv12 and Attention-MobileNetV2, developed for the autonomous inspection and damage detection of power transmission line insulators. The experimental evaluation is structured into three primary phases. First, the training dynamics and learning convergence of the models are analyzed to verify the effective mitigation of overfitting. Second, the end-to-end performance of the hybrid system is rigorously evaluated on the test dataset using comprehensive metrics, including IoU, classification precision, computational memory footprint, and inference speed. Finally, the empirical findings are benchmarked against established state-of-the-art architectures, followed by a critical discussion of the proposed method’s operational advantages and inherent limitations.

All training and testing procedures for the proposed models were executed on a high-performance Graphics Processing Unit (GPU) workstation to accelerate computationally intensive tensor operations. Throughout the training phase, rigorous hyperparameter optimization was employed to guarantee stable weight updates and facilitate convergence toward the global optimum, thereby preventing the models from stagnating in local minima. The detailed technical specifications of the hardware and software environments utilized for these experiments are summarized in Table 3. Concurrently, the final optimized hyperparameters configured for both the detection and classification stages are outlined in Table 4.

### 4.1. Object Detection Module YOLOv12n Test Results

Serving as the primary and most critical stage of the hybrid architecture, the YOLOv12n model directly dictates the downstream performance of the entire system. To exclusively evaluate its bounding box generation efficacy, an independent performance analysis was conducted on 197 test images, isolating the detection phase from the subsequent classification module. Inference testing was executed utilizing an NVIDIA T4 GPU. A structural summary and the overarching performance metrics of the model are outlined in Table 5, while a detailed class-wise analysis is presented in Table 6.

The paramount objective of this system is to ensure no insulator discs remain undetected within the field of view. As detailed in Table 6, the Recall metric for the “insulator disc” class reached 0.967, indicating that 96.7% of the target discs were successfully localized. Furthermore, the mean Average Precision at an IoU threshold of 0.50 (mAP@50) achieved 0.98. Under the more stringent mAP@50-95 metric, which evaluates the precise tightness of the predicted bounding boxes against the ground truth, the model yielded a robust score of 0.828. This high spatial precision guarantees that the extracted ROI patches forwarded to the secondary classification network are optimally centered and effectively isolated from background noise. Finally, despite a significant quantitative disparity between “insulator disc” and “insulator string” instances within the dataset, the comparable mAP scores across both classes demonstrate the model’s inherent resilience to class imbalance.

### 4.2. Classifier Stage Attention-MobileNetV2 Test Results

Serving as the primary diagnostic core of the proposed hybrid architecture, the Attention-MobileNetV2 classification model was evaluated independently of the object detection phase using a dedicated test dataset comprising 3090 insulator images. The model’s performance was analyzed concerning both its class-wise diagnostic capabilities and its overall computational efficiency, specifically regarding inference throughput and memory footprint. The resulting class-specific performance metrics for this classification stage are summarized in Table 7.

The developed classification model achieved a perfect recall score of 1.0 for both the broken and flashover damage classes. This outcome demonstrates that all defective discs within the test set were successfully identified, underscoring the system’s robust diagnostic reliability. Furthermore, a precision score of 1.0 for the normal class indicates that the model never misclassifies a healthy disc as defective. Conversely, the precision score of 0.972 for the fault categories implies that the model operates with a conservative safety margin; it occasionally flags highly ambiguous normal discs as potentially defective to absolutely minimize false negatives. Although normal samples constitute 82% of the test dataset, the high F1-Scores (0.986) achieved for the minority fault classes confirm that the integration of the Focal Loss function and the Convolutional Block Attention Module (CBAM) effectively mitigates the inherent class imbalance. Finally, the hardware efficiency and computational costs associated with this classification model are summarized in Table 8.

As detailed in Table 8, the model features a compact architecture, comprising merely 1.82 million parameters and requiring a minimal storage footprint of 7.08 MB. This lightweight profile facilitates direct deployment on resource-constrained UAV embedded systems, thereby circumventing the reliance on high-cost server infrastructures. Furthermore, the model achieves a high inference throughput of 183.95 FPS. This performance confirms the system’s capability to execute seamless, real-time diagnostic analysis in the field and provides sufficient computational bandwidth to process multiple camera streams simultaneously.

### 4.3. Performance Results of the Proposed Integrated Hybrid System

This section presents the end-to-end performance results of the proposed hybrid system, which integrates the YOLOv12n object detection and Attention-MobileNetV2 classification modules within a cascaded architecture. To closely simulate real-world field conditions, the evaluation pipeline was designed to accept raw input imagery and directly output final diagnostic labels. This comprehensive testing phase was executed on a dedicated dataset comprising 197 images, requiring the system to autonomously detect, extract, and classify the target structures. The integrated system’s overall efficacy in localizing insulator discs and diagnosing their respective damage states is summarized in Table 9.

The empirical results detailed in Table 9 demonstrate that the proposed cascaded architecture incurs no performance degradation despite its dual-stage nature. Crucially, the system achieved a perfect recall score of 1.0 for the broken disc class, the most critical metric for energy infrastructure safety. This absolute sensitivity confirms the successful detection of all 112 structurally defective discs within the test set, effectively eliminating hazardous false negatives. Furthermore, the overarching system accuracy of 99.71% robustly validates the high reliability and operational readiness of the proposed hybrid framework.

**Figure 5:**
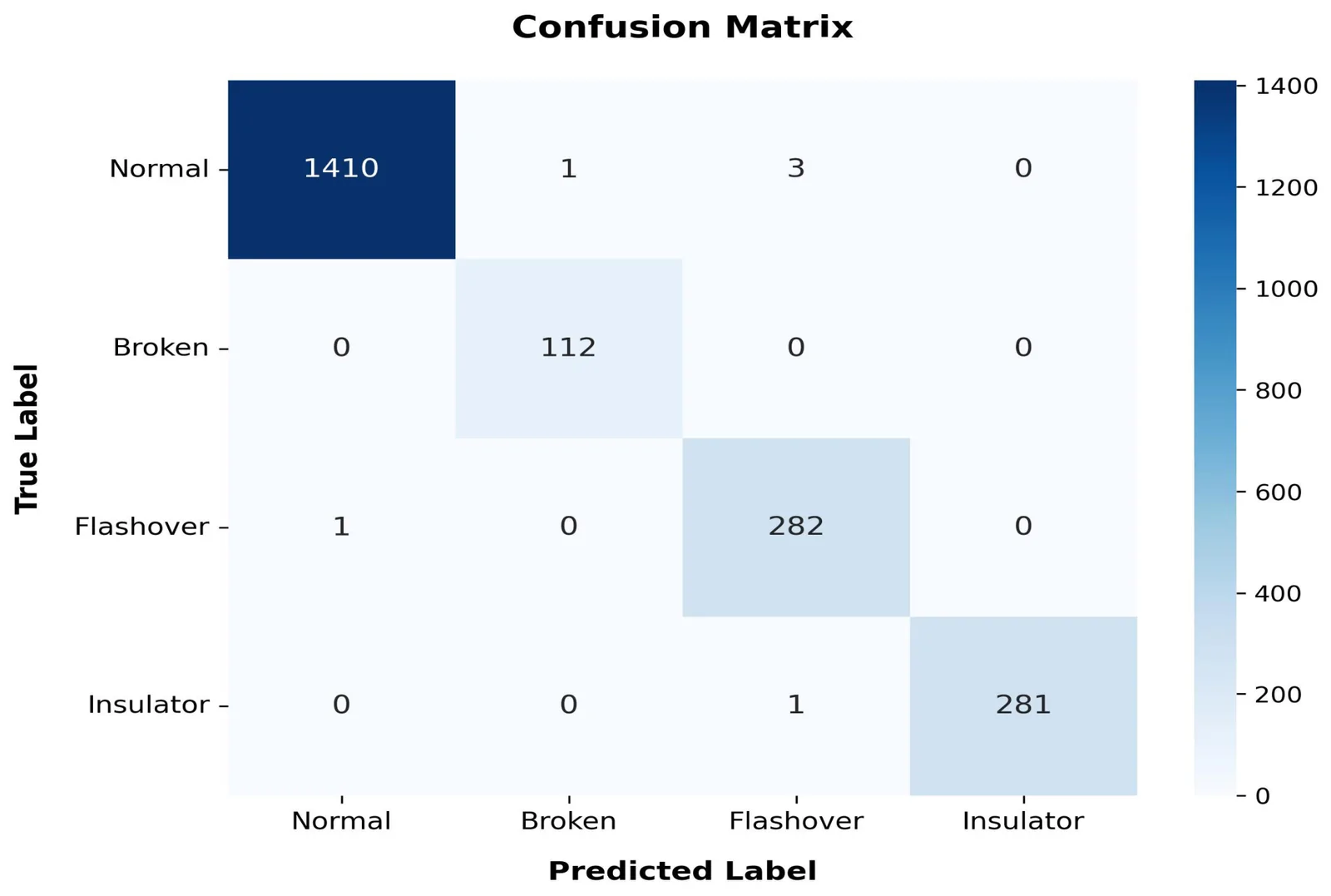
Hybrid system confusion matrix result.

Figure 5 presents the integrated confusion matrix for the proposed model. The dense concentration along the main diagonal visually corroborates the system’s exceptional discriminative accuracy of 99.71%. Notably, the “broken disc” row exhibits zero false negatives, reaffirming the system’s flawless detection capabilities for critical structural failures. Out of 2091 total test samples, merely six misclassifications occurred. The majority of these anomalies involved predicting “normal” discs as possessing “flashover damage,” demonstrating that the system operates with a deliberate and conservative safety margin, erring on the side of caution in highly ambiguous scenarios. Moreover, the absence of cross-confusion between the “flashover damage” and “broken” classes verifies the efficacy of the integrated CBAM in isolating distinct defect morphologies. The cumulative hardware footprint and computational overhead of the developed system are summarized in Table 10.

**Figure 6:**
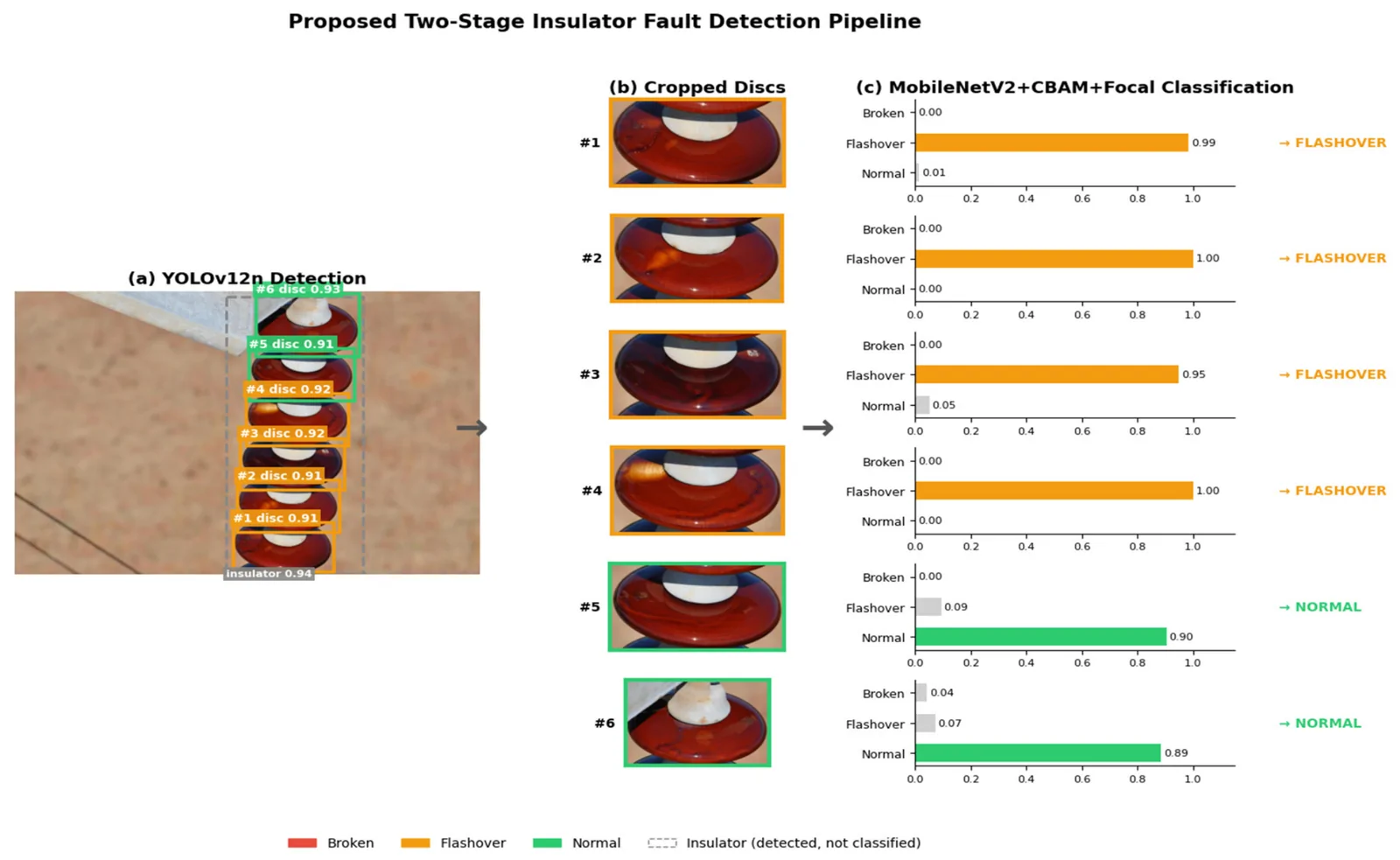
Detection and classification results of the proposed hybrid system on the Lewis dataset.

**Figure 7:**
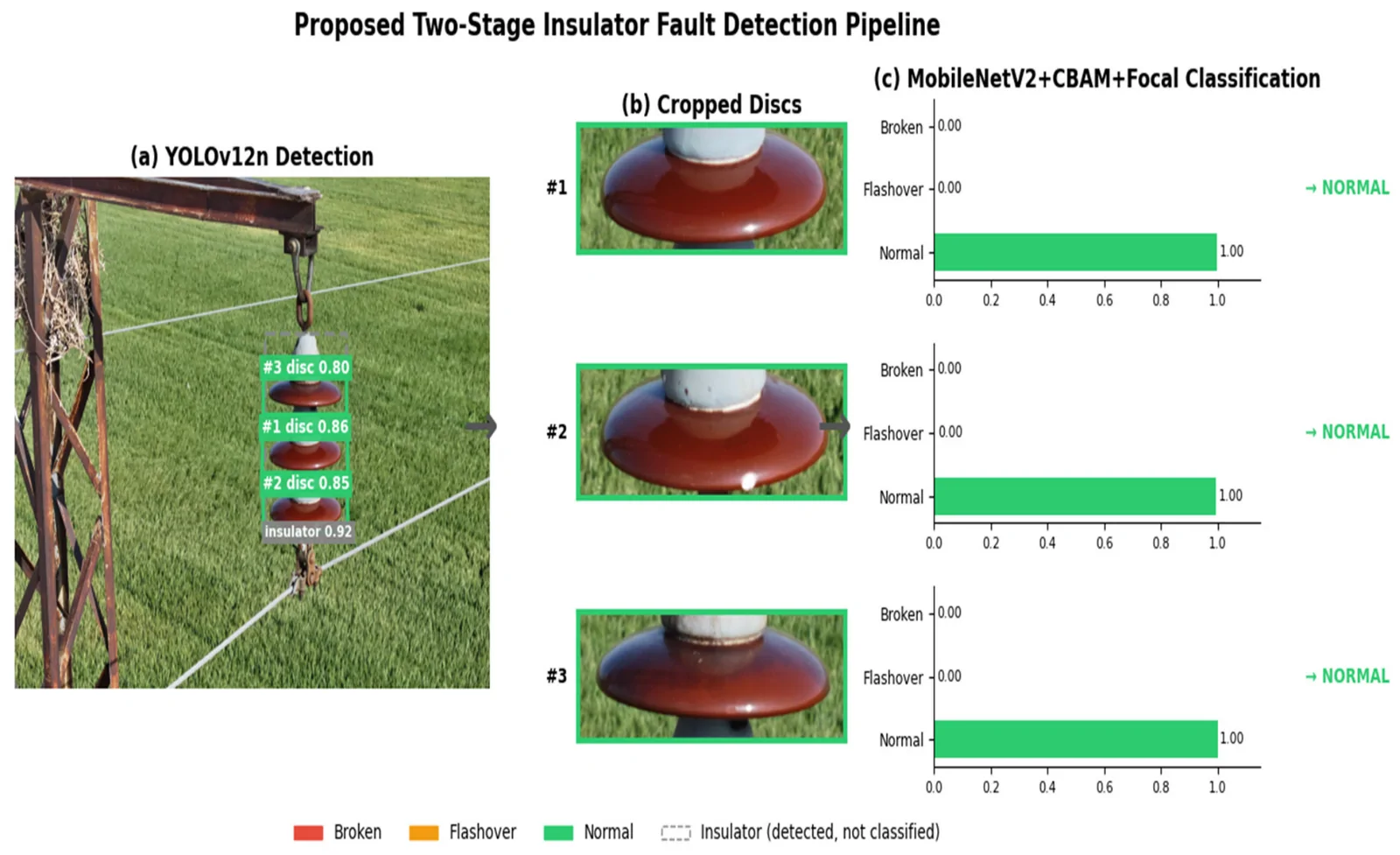
Detection and classification results of the proposed hybrid system on our dataset.

As outlined in the quantitative analysis, the cascaded system maintains a compact profile, comprising approximately 4.4 million total parameters and requiring a minimal storage footprint of only 12.45 MB. Coupled with an end-to-end inference throughput of 7 FPS, these metrics conclusively demonstrate that the proposed architecture can be deployed and efficiently operated within the severe storage, memory, and computational constraints characteristic of UAV-based embedded platforms. The hybrid system outputs on the Lewis dataset are presented in Figure 6. The corresponding hybrid system outputs on our dataset are presented in Figure 7.

## 5. Ablation Study

To comprehensively evaluate the performance gains of each optimization module in the proposed hybrid architecture, ablation experiments were conducted. These experiments aimed to assess the individual contributions of the secondary classification stage (MobileNetV2) and the integrated attention and loss mechanisms (CBAM + Focal Loss) across two different datasets: the public Lewis Dataset and our enriched field dataset (“Our Dataset”). The experiments were built upon the baseline YOLOv12n object detection model, progressively integrating the classification modules to quantify their impact on accuracy, precision, recall, and F1-score. The specific ablation experiment results are presented in Table 11.

First, the baseline YOLOv12n model was evaluated independently. On the Lewis dataset, it achieved an accuracy of 0.7860 and an F1-score of 0.7148. On Our Dataset, it demonstrated stronger initial performance with an accuracy of 0.8680 and an F1-score of 0.8769. This baseline establishes the capacity of a standard single-stage detector before any secondary classification refinement is applied.

In the second phase, the cascaded structure was introduced by appending the standard MobileNetV2 classifier to the YOLOv12n backbone. When tested on Our Dataset, this two-stage integration resulted in a substantial performance boost, increasing accuracy to 0.9224 and the F1-score to 0.9224. This indicates that decoupling the detection and classification tasks allows the network to preserve fine structural details extracted from complex backgrounds. Interestingly, on the Lewis dataset, while overall accuracy improved to 0.8531, the precision, recall, and F1-score (0.6074) experienced a decline. This anomaly suggests that applying a secondary deep classifier on a dataset potentially lacking sufficient background diversity or high-resolution complex features might lead to overfitting on the dominant classes, thereby reducing the recall for minority defect classes.

Finally, to address class imbalance and enhance the model’s sensitivity to subtle fault features, the CBAM attention module and Focal Loss function were integrated into the MobileNetV2 classifier. On Our Dataset, this final configuration achieved an exceptional accuracy of 0.9948 and an F1-score of 0.9948, marking a significant improvement across all metrics compared to the standard two-stage model. The CBAM successfully suppressed irrelevant background noise, while the Focal Loss effectively directed the network’s learning capacity toward the challenging, infrequent defect classes. Conversely, on the Lewis dataset, these additions maintained performance at a similar level to the standard MobileNetV2 (Accuracy: 0.8571, F1-Score: 0.6033).

These ablation results conclusively demonstrate that the proposed enhancements—specifically the two-stage hierarchical design coupled with CBAM and Focal Loss—are highly effective for real-world, complex field conditions as represented in Our Dataset. They validate that the proposed hybrid architecture significantly outperforms standard single-stage detection when tasked with identifying millimeter-sized defects in challenging and diverse environments.

## 6. Comparison with Related Work

This section comparatively analyzes the performance of the proposed hybrid deep learning model against that of standard architectures and architectures in the recent literature. A comparative analysis with recent studies is presented in Table 12. The literature indicates a trend toward hybrid approaches when single-stage models prove insufficient. However, existing two-stage and hybrid frameworks often face practical limitations regarding computational load and hardware requirements. For instance, Zhang et al. [15] proposed a sequence transduction approach utilizing Oriented R-CNN for detection and a DETR-like Transformer module for patch comparison. While the Multi-Head Self Attention mechanism captures global relationships effectively, the massive parameter count of the ResNet50 backbone and Transformer structure makes it impractical for resource-constrained edge devices like UAVs. Similarly, the STID framework by Han et al. [16] employs YOLOv11n-seg for masking and Reverse Distillation for anomaly detection. Although this completely suppresses background noise, its rigid cascaded structure means that any minor segmentation error directly results in false positives or false negatives. Furthermore, Sun et al. [17] combined YOLOv5 with traditional image processing techniques such as Gamma correction and unsharp masking. While these filters reduce low-light and motion blur issues, traditional thresholding loses its generalization capability under variable weather conditions.

The system proposed in this study adopts a hierarchical philosophy similar to in recent literature but prioritizes maximum efficiency by utilizing lightweight backbones, specifically YOLOv12n and MobileNetV2, optimized for edge devices. As a point of comparison, Souza et al. [26] proposed a “Hybrid-YOLO” combining YOLOv5x and ResNet-18, which achieved high accuracy but relied on computationally heavy architectures that increase energy consumption and latency—significant drawbacks for UAV-based deployment. The proposed method effectively resolves the well-documented speed–accuracy dilemma found in traditional models. Previously, researchers struggled to balance these two metrics; for example, Zhao et al. [27] achieved a high mAP using an Improved Faster R-CNN, but the model remained computationally cumbersome, whereas their IDD-YOLO model prioritized speed at the cost of a severe drop in accuracy. Compared to the Improved Faster R-CNN, which required 118 MB of memory and achieved a flashover AP of 74.7, the proposed hybrid model reduces memory usage by 89.4% down to just 12.45 MB. Despite this smaller footprint, it delivers a vastly superior flashover AP of 0.99 and an overall F1-score of 0.99.

In terms of metric analysis, the proposed framework achieves a near-perfect 99.0% overall mAP. This presents a stark contrast to the Transformer model by Zhang et al. [15], which only reached a 70.2% mAP50 despite running on powerful dual NVIDIA A30 GPUs, demonstrating a struggle to learn effectively from small or imbalanced datasets. The sub-class performance further highlights this advantage; while a standalone YOLOv12n model yielded AP scores of 0.88 and 0.87 for broken and flashover classes, respectively, the specialized classification layer of the hybrid structure elevated both to 0.99. A detailed comparison of the insulator and disc classification performance is presented in Table 13. In contrast, Zhang et al.’s model managed only 64.8% for breakage and 75.5% for missing caps [15].

Finally, detecting small, shape-related defects remains the ultimate benchmark. While models like Serikbay et al.’s DS-YOLOv11n recently set a strong standard for embedded systems, single-stage models still risk missing minute details [26]. Thanks to an integrated CBAM that filters background noise, the proposed system achieved a flawless 1.0 recall for the most challenging broken class. This zero miss rate and a 99% F1-Score demonstrate the superiority of the attention mechanism over existing hybrid structures. Ultimately, by combining maximum defect sensitivity with an end-to-end processing speed of approximately 7 FPS and a lightweight 12.45 MB footprint, this architecture achieves the optimal balance required for resource-constrained UAV deployment.

## 7. Conclusions

In this study, a lightweight and high-performance hybrid deep learning model combining YOLOv12n and CBAM-integrated MobileNetV2 architectures is proposed for the autonomous and real-time detection of insulator faults in power transmission lines, tailored for UAV applications. By integrating the computational speed advantages of object detection networks with the precision of attention-based classifiers, the developed cascade system achieved a 100% recall rate in detecting “Broken Disc” defects, the most critical fault for power grid security, and an overall accuracy of 99.71%, thereby successfully overcoming the traditional speed accuracy trade-off.

With a compact model size of only 12.45 MB and an end-to-end processing speed of ~7 FPS, the proposed architecture delivers an optimal solution for industrial field inspections, maintaining a high fault detection performance (F1-Score: 99%) even under the constrained hardware resources of autonomous UAV platforms. Future work will focus on enhancing the model’s robustness against adverse weather conditions using synthetic data generated via GANs, integrating thermal imaging for the detection of heating-related faults, and conducting real-world flight tests on embedded edge processors.

Although the proposed model demonstrates high efficiency and superior accuracy (e.g., Flashover AP of 0.99) for edge-device deployment, it is important to acknowledge certain limitations in real-world applications. First, when deployed on UAV platforms, rapid camera movements or sudden aerodynamic shifts can introduce severe motion blur, which may temporarily degrade real-time detection performance. Second, extreme environmental conditions—such as heavy fog, thick snow, or dense smoke—can severely obscure visual features, limiting the model’s reliability in such scenarios. Finally, while the model is highly optimized with a footprint of 12.45 MB, deploying it on ultra-low-power microcontrollers with even stricter hardware constraints may require additional model compression techniques, such as heavy quantization or pruning. Future research will focus on addressing these challenges to further enhance robustness in adverse operational environments.

## Figures and Tables

**Table 1:** Distribution hybrid data set.

Data Set	Source	Images	Objects
Train	[20]	1118	13,271
	Original	253	2627
Val.	[20]	316	3715
	Original	75	819
Test	[20]	166	1926
	Original	31	276
Total		1959	22,634

**Table 2:** Comparison of classification models’ performance and resource usage.

Model	Acc.	F1	FPS		Param.	Model Size
			Tesla T4	CPU		
EfficientNet	99.51	0.995	233.7	13.5	4.01 M	15.67
MobileNetV2	99.16	0.991	216.0	22.3	2.23 M	8.81
ResNet-18	98.38	0.983	252.4	10.8	11.18 M	42.80

**Table 3:** Hardware and software configuration.

Component	Feature/Version
CPU	Intel Core i7-8750H @ 2.20 GHz
GPU	NVIDIA Tesla T4 (16 GB GDDR6 VRAM)
RAM	12 GB
Operating System	Ubuntu 22.04 LTS
Software	PyTorch 2.0.1 + CUDA 11.8
Libraries	Ultralytics 8.3, Timm 0.9, OpenCV 4.8

**Table 4:** Hyperparameters used in model training.

Hyperparameters	YOLOv12n	Attention-MobileNetV2
Input	640 × 640 pixel	224 × 224 pixel
Iteration	50	20
Batch Size	16	32
Loss Function	Box + DFL + Cls Loss	Focal Loss (γ = 2.0, α = 1)

**Table 5:** YOLOv12n model structural summary and speed performance.

Feature	Value
Model Architecture	YOLOv12n
Number of Layers	159
Number of parameters	2,557,118
Processing Load (GFLOPs)	6.3
Theoretical Speed (FPS)	~104.85 FPS

**Table 6:** YOLOv12n model class-based detection performance.

Class	Sample	P	R	mAP@50	mAP@50-95
Disc	1899	0.93	0.96	0.98	0.832
Insulator	303	0.95	0.96	0.98	0.824
Avg.	2202	0.94	0.97	0.98	0.828

**Table 7:** Results of the Attention-MobileNetV2 classifier.

Class	Precision	Recall	F1	Sample
Broken	0.972	1.000	0.986	210
Flashover	0.972	1.000	0.986	346
Normal	1.000	0.994	0.997	2534
Avg.	0.995	0.995	0.995	3090

**Table 8:** Hardware resource usage and speed test of the Attention-MobileNetV2 classifier.

Performance Metric	Value
Accuracy	99.48
mAP	0.9997
Total Parameters	1.82 M
Model Size	7.08 MB
T4 GPU FPS	183.95

**Table 9:** Classification performance of hybrid system.

Class	Precision	Recall	F1	Sample
Broken	0.9912	1	0.9956	112
Flashover	0.9860	0.9965	0.9912	283
Normal	0.9993	0.9972	0.9982	1414
Insulator	1	0.9965	0.9982	282
Avg.	0.9972	0.9971	0.9971	2091

**Table 10:** Architectural features and resource utilization of the hybrid system.

Modul	Parameter	Model Size	Layer
YOLOv12n	2,568,438	5.24 MB	272
MobileNetV2	1,825,573	7.20 MB	216
Hybrid System	4,394,011	12.45 MB	488

**Table 11:** Ablation study results demonstrating the contribution of each component in the proposed model.

Dataset	Model	Accuracy	Precision	Recall	F1-Score
Lewis Dataset	YOLOv12n	0.79	0.67	0.77	0.71
	YOLOv12n + MobileNetV2	0.85	0.64	0.69	0.61
	YOLOv12n + MobileNetV2 + CBAM + FocalLoss	0.86	0.63	0.66	0.61
Our Dataset	YOLOv12n	0.87	0.89	0.87	0.88
	YOLOv12n + MobileNetV2	0.92	0.92	0.92	0.92
	YOLOv12n + MobileNetV2+ CBAM + FocalLoss	0.99	0.99	0.99	0.99

**Table 12:** Comparative Analysis with Recent Studies.

Study	Architecture	Strengths	Limitations
Zhang et al. [15]	Oriented R-CNN + DETR-like Transformer	Captures global contextual relationships using Multi-Head Self-Attention	Very high computational complexity, large parameter count, requires dual NVIDIA A30 GPUs, limited performance on small/imbalanced datasets (70.2% mAP50)
Han et al. [16]	YOLOv11n-seg + Reverse Distillation (STID)	Removes background noise effectively through segmentation	Segmentation errors directly propagate to anomaly detection, increasing false positives/negatives
Sun et al. [17]	YOLOv5 + Traditional Image Processing	Improves low-light and motion-blur images	Hand-crafted preprocessing lacks robustness under varying weather conditions
Souza et al. [26]	Hybrid-YOLO (YOLOv5x + ResNet-18)	High detection accuracy	Heavy backbone increases latency, energy consumption, and memory usage
Zhao et al. [27]	Improved Faster R-CNN/IDD-YOLO	Faster R-CNN provides high accuracy; IDD-YOLO improves inference speed	Faster R-CNN is computationally expensive (118 MB), while IDD-YOLO sacrifices accuracy for speed
Serikbay et al. [26]	DS-YOLOv11n	Strong embedded performance	Single-stage detector may miss fine structural defects
**Proposed Method**	YOLOv12n + MobileNetV2 + CBAM + Focal Loss	Lightweight (12.45 MB), 99.0% mAP, 0.99 F1-score, 1.00 Recall (Broken), ~7 FPS, suitable for UAV edge deployment	Two-stage pipeline introduces a small additional inference step

**Table 13:** Comparison with Insulator and Disc classification mode.

Model	AP50	AP-Normal	AP Broken	AP Flashover	AP Insulator	F1	Precision	Recall
R50_FPN_3x	0.86	0.71	0.48	0.56	0.81	0.85	0.86	0.85
R_50_C4_3x	0.89	0.73	0.49	0.64	0.76	0.85	0.89	0.81
R_101_FPN_3x	0.90	0.75	0.49	0.66	0.80	0.90	0.90	0.89
X_101_3x	0.90	0.73	0.47	0.63	0.77	0.89	0.90	0.88
YOLOv5-x	0.87	0.85	0.90	0.77	0.97	0.85	0.85	0.86
YOLOv8-x	0.93	0.90	0.94	0.87	0.99	0.88	0.90	0.87
YOLOv10-x	0.89	0.88	0.90	0.81	0.99	0.86	0.87	0.85
YOLOv11-x	0.93	0.90	0.94	0.87	0.99	0.89	0.88	0.90
YOLOv12-x	0.94	0.94	0.94	0.90	0.97	0.89	0.88	0.90
YOLOv12-n	0.92	0.92	0.88	0.87	0.98	0.88	0.88	0.88
**Proposed Model**	0.99	0.99	0.99	0.99	1	0.99	0.99	0.99

## Data Availability

The data supporting the findings of this study are available from the corresponding author upon reasonable request. The dataset is not publicly available due to restrictions related to the source datasets and the use of field-collected UAV imagery.
